# Facing the rising sun: Political imagination in Black adolescents’ sociopolitical development

**DOI:** 10.3389/fpsyg.2023.867749

**Published:** 2023-02-22

**Authors:** Edward D. Scott, Johari Harris, Chauncey D. Smith, Latisha Ross

**Affiliations:** ^1^Graduate College of Social Work, University of Houston, Houston, TX, United States; ^2^Bagwell College of Education, Kennesaw State University, Kennesaw, GA, United States; ^3^School of Education and Human Development, University of Virginia, Charlottesville, VA, United States; ^4^Youth-Nex, The UVA Center to Promote Effective Youth Development, Charlottesville, VA, United States

**Keywords:** Black adolescents, youth activism, political theory, sociopolitical, imagination (psychology)

## Abstract

Black adolescents occupy one of the most precarious and marginalized social locations of society, yet they remain vigilant against oppression. Indeed, Black youth have a vast history of political action and activism around domestic and global issues. Existing scholarship frequently examines the sociocultural and cognitive factors associated with Black adolescents’ political and civic engagement and related outcomes. Lost in these interrogations is an examination of the psychological processes that undergird adolescents’ sociopolitical visions. To address this gap, this conceptual analysis examines political imagination and its role in Black adolescents’ sociopolitical development. Political imagination is the cognitive space and process where people consciously distance the present moment to engage, explore, examine, and (de)construct sociopolitical worlds or realities.

## Introduction

Black adolescents occupy one of the most precarious social locations of society, yet they remain vigilant against oppression. Black youth have a vast history of political engagement and activism where they have addressed a multitude of issues, such as educational equity, environmental justice, juvenile justice reform, and school police abolition (Braxton, 2016; Anyiwo et al., 2018; Turner, 2018; Franklin, 2021). As righteous rage and demands for social transformation in the United States and global context continue, Black youth are watching, processing, learning, and responding to these situations with their futures in mind.

Concerningly, society mainly focuses on politically enfranchised adults’ reimaginings of the sociopolitical landscape (Bertrand et al., 2020; Clay and Turner, 2021; see also Rodela and Bertrand, 2021). This is a shortsighted approach that limits social progress. Adults are not the only persons capable of reimagining, nor the only persons for whom reimagining is consequential. Indeed, many youth-serving organizations have expressed commitments to reimagining their youth work with focuses on social justice, diversity, equity, inclusion, and antiracism. Yet, during the reimagining, those institutions often fail to acknowledge, cultivate, and leverage Black youths’ sociopolitical voices, visions, and agency. They ignore a simple, continuous truth: Black youth have politics.^1^

As various stakeholders move to reimagine many aspects of society, Black youths’ political visions must be made more central to that restructuring. This paper’s purpose is to situate the concept of political imagination (see Wiley, 2016) within developmental science literature to further nuance academic discourse and inform entities supporting Black youth’s political engagement and activism, particularly Black youth in adolescence. Here, political imagination is the cognitive process and space where people consciously distance the present moment to engage, explore, examine, and (de)construct sociopolitical worlds or realities. The political imagination of Black adolescents is important and warrants particular attention, given their unique status within the United States and global contexts where they often are marginalized under anti-Black racism and youth-targeted ageism (Sallah et al., 2018; Velez and Spencer, 2018). Drawing on the research from political theory and psychological science, we integrate political theory’s concept of political imagination into Sociopolitical Development Theory (SPD; Watts et al., 2003), highlighting its implications for Black adolescents’ transition from critical social analysis (CSA) to critical action (CA).

There are three premises underpinning our conceptualization of Black adolescents’ political imagination within the context of SPD: (a) imagination’s relevance to adolescence, (b) imagination’s relevance to politics and transformative action, and (c) adolescence’s relevance to politics and transformative action. First, we briefly present an overview of SPD to introduce the central framework and guide readers to our more particular focus on Black adolescents’ transitions from CSA to *CA.* Then, we discuss the imagination as it pertains to Black adolescent development and transformative political action, which is necessary given the current disconnect between imagination and adolescent development research (see Appendix A). Lastly, we address the dynamics between youth and adults directly by providing examples about engaging political imagination across various developmental settings and include recommendations for future research. Ultimately, this is a call to affirm Black adolescent political imagination.

## Sociopolitical development theory

The SPD model was developed through research with Black youth with a focus on the recursive relationship between critical analysis of social conditions and critical action—liberatory praxis (Watts et al., 1999, 2003). SPD incorporated tenets of liberation psychology into developmental psychology during adolescence by focusing on “exposing social injustice, creating just societies, promoting self-determination and solidarity with others, and ending oppression (and healing its effects)” (Watts and Guessous, 2006, p. 60). SPD has been refined into a developmental model and undergirded by four propositions: (a) analysis of authority and power are central to informing one’s critiques of extant political structures; (b) a sense of agency is critical to motivating political action; (c) action requires opportunity; and (d) commitment and action are desired developmental outcomes.

While SPD frames the developmental process well, SPD literature often focuses on *either* the action element *or* the analysis element of the model, usually placing more emphasis on the reflection. Diemer et al. (2020) highlight this bifurcation in their call for SPD and critical consciousness researchers to recenter action in this work. While the call for more action-focused research is warranted, there also is a need to ensure scholarship is not solely focused on youth resistance but also considering their freedom vision and wellbeing. Critical action ought not only be driven by what youth want to be *liberated from* or *free of*, but also must consider—even center—what the youth want to be *liberated for,* what they want to be *free to do* or *be* or *experience.* This is best understood when we consider the worlds envisioned between critical reflection and critical action.

### Political imagination: Bridging critical social analysis to critical action

Political imagination provides a way to think about that space between CSA and critical action—the bridging. We argue that Black adolescents form and refine their vision of a just society and liberation through political imagination during SPD. The figments of Black adolescents’ political imagination are the result of being in their particular sociopolitical location (i.e., Black youth in adolescence) at a particular moment in time and critically reflecting on their social condition (Hope and Bañales, 2019). Informed by social factors, political imagination is the process in which Black youth explore their social justice interests, establish their demands, experiment with revolutionary strategies, and conspire for exodus or revolt. Black adolescents outline the intentions and goals that orient their social justice leadership during political imagination. Black adolescents’ political imagination is enabled when they suspend (a) presentism, (b) determinism, and (c) disbelief. Then, they can engage the imaginative field in the mind that is the ground of their new world (Root-Bernstein, 2013). Ideally, imagining would be approached conscientiously as to also avoid replicating or merely evolving the status quo. In doing so, Black youth can build and experience the world as they want it to be.

Political imagination is a distinct yet interrelated concept from CSA or critical reflection. Watts et al. (2003) wrote, “Liberation requires vision—a transition from critique to creativity. Critique reveals the need for new ideas and action, but creativity is required to envision a better cultural and moral order” (p. 187). Critical Social Analysis (CSA) relates to political imagination because it is a cognitive rupture that leverages both past and the present to launch Black youth’s minds to a different reality (imaginative field). CSA is the point in the SPD process where Black youth can benefit the most from (re)membering the stories of Black youth activists’ wins, critically reflecting on the tradition of social justice movements led by Black youth.

Connecting political imagination to SPD does not fundamentally create a new theory or model, but rather it deepens and enhances the existing one. Political imagination is a tool Black youth (can) use both during and after critically analyzing social structures to then inform their subsequent action. Imagining allows Black youth to reorient themselves, their consciousness and understanding, to time and possibility. For example, Black adolescents can look back and recognize the miseducation of their community and its link to the present conditions of Black people. One’s understanding of and connection to community is one of the most influential elements of societal development (McBride, 2005). Developing a sense of group identity, including racial or ethnic identity, also is a key part of adolescent development (Anyiwo et al., 2018). Relatedly, Black youth also can look forward. Political imagination allows Black youth to practice centering and creating *possibility*, unbound by present reality while critically aware. In fact, it is a critical awareness of reality that drives Black adolescents to procure the alternative and recognize it as it materializes. Several Black scholars in various disciplines (see Hooks, 2000; Dawson, 2001; Kelley, 2002) have mentioned imagination and similar concepts in their discussions of social change, yet here we focus specifically on Black adolescents and their political imagination’s contributions to that endeavor.

## On Black adolescence and political imagination

Growing up Black in a relentlessly anti-Black and Eurocolonized world means Black adolescents also are regularly exposed to discrimination, which has detrimental health effects (Sellers et al., 2006; Hope et al., 2018, 2019; Seaton and Iida, 2019; DeBower et al., 2021). The developmental attributes of adolescence can be leveraged for Black youth to explore their sociopolitical interests and create a new world through imagination. Imagination is the process of mentally transcending time, space, and circumstance to examine past histories, test ideas, and create alternative worlds (Taylor, 2013; Hawlina et al., 2020). Scholars (Zittoun and Cerchia, 2013; Kind, 2016) generally agree that imagination is best conceptualized as a process, rather than simply its products. The imagining mind is both fully conscious of the present and able to move across time. The imaginative process allows individuals to engage and exploit the temporal space between *what is* and *what could be*, between present and possible (Zittoun and Cerchia, 2013).

From a philosophical standpoint, imagination’s content has been most simply understood as a reflection of the condition of one’s consciousness (Kind, 2016; Zittoun and Glăveanu, 2017). There is a dearth of research that focuses on imagination during adolescence (Gajdamaschko, 2005, 2006), and imagination also is an essential yet understudied concept in politics (McBride, 2005; Wiley, 2016). Extant research on imagination predominantly focuses on imaginative processes and outcomes of youth from infancy to middle childhood (Root-Bernstein, 2013; Taylor, 2013; Weisberg, 2016). Adolescents maintain their ability to imagine beyond childhood, but nurturing their imaginative capacity typically is not prioritized by society (Gajdamaschko, 2005; Eckstein et al, 2012). To the contrary, scholars (Zittoun and Cerchia, 2013) note how the proclivity for imagination often is socialized out of adolescents.

Supporting Black adolescents’ SPD requires providing the opportunity for Black youth to support one another in critically interrogating the past and present to imagine the possible. Critical political discourse informs the political imagination. Black adolescents’ political imaginations are the nurturing soil for the seeds of critical hope (Duncan-Andrade, 2009) that mature and manifest new worlds. With adolescence comes an increase in both personal and sociopolitical awareness, which often catalyzes a personal investment in combating injustice for Black youth (Anyiwo et al., 2020; Plummer et al., 2022). Adolescence often brings about the social expectation of being *logical* and *concrete* in one’s thinking, reinforcing those *framings* of cognition as the basis of producing pragmatic solutions to common problems (Gajdamaschko, 2005; Zittoun and Cerchia, 2013). The limited research on adolescent imagination generally notes the influence of sociocultural context, cultural knowledge, or history (Gajdamaschko, 2005, 2006; Zittoun and Cerchia, 2013).

Indeed, Black adolescents are well situated to capture and digest the nuances of the sociopolitical landscape because of their unique social location and cultural background. Cammarota (2011) leverages W. E. B. Dubois’ concept of *second sight* which helps frame the heightened social awareness that Black youth experience. The term second sight gives language to Black folks’ vision of and for self beyond subjection to oppression; they simultaneously see the realities of oppressive systems and their commitment to experience life without subjugation (Cammarota, 2011). This second sight compounds the heightened social awareness and sensitivity of adolescence, suggesting that contextual factors would have a particularly significant influence on Black adolescents’ political imaginations. Increased capacity for abstraction also helps Black adolescents understand that *the past* is continuously being made *at* the present, so the trajectories set in previous historical moments can be changed, which stands to alter outcomes of the future. These understandings reflect their ability to suspend presentism and determinism. Suspending disbelief creates an unobstructed stream of thoughts where the youth can freely interact with the imaginary worlds they create. Youth draw on complex linguistic, reasoning, semiotic, historical, social, and cultural knowledge and skills to navigate the here-and-now (i.e., to manage the present); they also leverage those knowledges and skills to regulate their relationship to the here-and-now (i.e., to leave the present while still having those resources; Zittoun and Cerchia, 2013).

Zittoun and Cerchia (2013) wrote, “Imagination always seems to open a different space, or a different modality of thinking, which eventually terminates when the person ‘comes back’ to reality. Imagination can be seen as an excursion” (p. 306). They refer to imagination as a mental loop out of the present. People begin imagining when something disrupts or ruptures their flow of thinking and attachment to the temporal present (Zittoun and Cerchia, 2013). The rupture can be chosen by the youth as well as imposed by a circumstantial occurrence or other individuals (Zittoun and Cerchia, 2013). Zittoun and Cerchia’s loop concept presupposes certain ontological assumptions about temporality and consciousness, yet there also is a need to recognize the cognitive skills imagining requires: (a) Suspending presentism; (b) Suspending determinism; and (c) Suspending disbelief.

Adolescence is a prime developmental opportunity to support and reinforce Black youths’ political imagination and engagement (Eckstein et al., 2012; Hope and Bañales, 2019; see also Walker et al., 2021). While adolescents are not old enough to engage in certain civic activities, such as voting, they are able to amplify their voices through other avenues, such as speaking at city council meetings, corresponding with governmental leaders, and protesting. Adolescence brings: (a) significant increases in sensitivity and awareness of social dynamics and rewards; (b) increases in their ability to think abstractly and understand complex ideas; (c) reduced inhibition regarding risks; and (d) increased desire to test social boundaries and explore social groups to cement their identity (Watts and Flanagan, 2007; Steinberg, 2011, 2014; Hope and Bañales, 2019).

Each of those developmental changes enhances Black youth’s ability to conceptualize complex social justice issues and imagine possibilities other than the current sociopolitical conditions. Desiring autonomy as well as relationships with individuals who share common interests and concerns is found among politically engaged adolescents, and this is reflected in the contemporary moment where Black youth are organizing communities in the movement for Black lives. When collectives of individuals desire sociopolitical change they: (a) form political communities, (b) organize around a particular issue or identity, (c) establishing a common understanding or collective consciousness, (d) imagine an alternative social and political order, and (e) act to reform or replace their current sociopolitical environment and the institutions therein (McBride, 2005). However, supportive contexts and relationships are important for counterbalancing the demands associated with adolescents’ developmental transitions which will be discussed further below.

## Affirming Black adolescents’ political imagination

### Framing spaces to engage black youths’ political imagination

There are two particularly helpful frameworks to guide how youth-serving organizations can best support Black youth in political imagination toward transformative, social-justice oriented action: Youthtopias (Akom et al., 2008) and Youthspaces (Goessling, 2020). Both of these concepts are rooted in the belief that youth have the capacity to positively contribute to society and their agency is most effectively enacted when environments are deliberately created with such in mind.

#### Youthtopias

Grounded in ongoing work in youth activist educational spaces, Akom et al. (2008) proposed a theoretical framework that centers youth’s skills, passion, knowledge, and overall assets “to lay the foundation for community empowerment and social change”: youthtopias. Youthtopias merge Youth Participatory Action Research (YPAR), Critical Race Theory, and critical media literacy all in the interest of promoting youth SPD. Critical Race Theory is an analytical lens that addresses the ubiquity and persistence of racism in every dimension of power in the United States and the broader Western world. Critical media literacy is another analytical, methodological, and pedagogical tool that engages participants in understanding, critiquing, and re-understanding all forms of media (Anyiwo et al., 2021). The tool aims to guide participants through uncovering truth as it relates to power in all forms of media and its consumption. Taken together with YPAR, youthtopias are spaces where Black youth: understand multiple intersecting oppressions, challenge traditions, co-construct and engage new possibilities, and commit to social justice (Akom et al., 2008).

#### Youthspaces

Goessling (2020) conducted a study to recount her work with youth to create *youthspaces*. Youthspaces are co-created spaces intentionally designed to facilitate critical inquiry about their lived experiences in creative arts and treating those creative arts *as* healing practices. The goal of these spaces should be to center the youths’ interest, issues, and priorities. This is done through the messages, teaching, artifacts, storytelling, affirmations, and modeling of collective-being in Black adolescents’ communities. Goessling’s (2020) youthspaces framework draws on both sociocultural theory and Freire’s concept of praxis for critical consciousness. In youthspaces, the deterministic outlook of trauma’s impact is rejected, and instead, youth are assumed capable of learning and acting in ways that leverage their personal knowledge and experience for their collective healing and social transformation. Contexts purposefully designed to promote Black youths’ sociopolitical development will focus on bolstering and refining their understanding(s) of the political dynamics and the role of their political action in instigating sociopolitical climate change.

### Supportive developmental contexts for political imagination

Black adolescents are most effectively aided in building a free(er) new world when in contexts that reinforce a fervent pursuit of liberation. However, it is antithetical to the liberation project to *force* adolescents to lead social justice movements (Cohen, 2010); there is dialectical tension of ethics in forcing people to be free. Instead, Black youth must be presented with opportunities that prompt political imagination and that privilege the youths’ freedom vision as the inspiration and compass for their transformative action. Those who support Black youth activists must affirm their efforts to push and advance our community in news ways (O’Donoghue and Strobel, 2007). Respectable resistance often is an expectation imposed on Black youth responding to injustice (Duncan-Andrade, 2009; Cohen, 2010; Harris, 2014; Clay and Turner, 2021). A recent example is found in critiques of the Black youth mobilizing as a part of the Movement for Black Lives who are castigated for being too radical, too de(con)structive. Black youth often are socially reprimanded for their political engagement (Cohen, 2010; Nummi et al., 2019). This reprimand certainly comes from some popular news outlets, but also from veteran mobilizers who subscribe to particular politics about direct action. In this respect, some within the mobilizing community have adopted a practice of policing Black youth political action that mirrors the government. When ideas from Black youth’s political imaginations are enacted, there often are positive sociopolitical consequences; affirming such reifies Black youth’s collective political efficacy and asserts their agency. The longstanding history of Black youth mobilization suggests that Black communities already consistently promote Black youths’ pursuit of freedom, yet it also is important to support youth in refining their freedom vision. Given this reality, both individual efforts and large-scale programming can and should be explicitly focused on facilitating Black adolescents’ early political engagement and SPD (Akom et al., 2008; Cohen, 2010). We attend to the particularities of efforts in key contexts of importance such as families, faith communities, youth-serving organizations, and schools, which are discussed below.

#### Families

Racial socialization is one mechanism by which parents might foster political imagination. Dimensions of racial socialization capture the ways in which parents prepare Black children for environments that are hostile to their racial ethnic group, and instill cultural knowledge, values, and pride, promotion of mistrust (e.g., encouragement to exercise caution in interracial relationships), and egalitarian values. Racial socialization has shown to be beneficial for a variety of outcomes for Black children. Healthy racial identity development (Huguley et al., 2019), academic success (Wang et al., 2020b), psychological adjustment (Wang et al., 2020a), and positive coping strategies (Anderson and Stevenson, 2019) have all been linked to parents’ racial socialization practices. Racial socialization also has benefits for Black youth’s sociopolitical development. Racial pride messages received from parents are associated with greater racialized critical social analysis (Lozada et al., 2017). Racial centrality is associated with civic engagement *via* Black consciousness, a construct inclusive of sociopolitical beliefs (Chapman-Hilliard et al., 2020). Scholars have begun to make the argument that racial identity and critical consciousness constructs overlap in youth’s critical reflection and likely develop in tandem (Mathews et al., 2020). Links between racial identity, youth activism, and civic engagement support this assertion (Hope et al., 2019). By extension, racial socialization, racial identity’s precursor (Neblett et al., 2009), shares in this overlap with the development of sociopolitical beliefs in Black youth as parent racial socialization predicted increases in Black youths’ critical analysis of systemic racism (Bañales et al., 2019b). These findings offer fertile ground for the socialization of political imagination.

Racial socialization is necessary to Black parenting practice. Black youth navigating racism and bigotry suffer racial stress and trauma, and racial socialization supports children’s coping processes (Anderson and Stevenson, 2019). However, what if we could imagine that Black youth did not have to navigate racism and bigotry? What would that look like? In part, it would look like some of what we have seen in extant Black political movements where Black youth imagine and work toward overcoming oppressive systems with the support and confidence of the adults around them. Black families serve as a conduit for political imagination-preparing change agents that are not shackled to broad American or White political imagination limitations and work to hold America accountable on the promise of freedom.

#### Schools

Schools present a rich context for adolescents to develop their political imagination. While many scholars have noted the many ways schools (Coles, 2016) fail Black youth, school contexts that consider adolescent’s developmental needs alongside school policies and practices that promote equity and inclusion can be sites of liberation (Cammarota, 2011; Love, 2019). Indeed, schools such as the Children’s Defense Fund Freedom Schools demonstrate the ways students develop critical consciousness and civic engagement skills through teacher practices and curricular materials (Jackson and Howard, 2014). Moreover, given the increasing amount of time adolescents spend within these spaces, it is a key site of socialization and their experiences within these spaces will affect a range of outcomes that extend far beyond academic achievement.

Given this reality, schools are fertile grounds to develop adolescents’ political imagination. Importantly, this can and should be done across levels (e.g., classroom level, school level, district level). For example, at the classroom level, given the centrality of the dialectic process among peers in the construction of Black youth’s political imagination, teachers can carve out space for this process to occur. In addition, given the importance of CSA in the process, teachers have the opportunity and responsibility to present students with meaningful, nuanced historical, social, and political content that provides insight into the issues of today (Bañales et al., 2019a). In addition, teachers can provide students with tangible research skills (e.g., navigating online, open source databases) necessary to engage in their own quest for answers. As noted earlier, it is an undue burden to make Black youth assume responsibility for social movements. Thus, teachers’ presence as co-conspirators can be much-needed support to adolescents. This can be as simple as allowing adolescents to express their ideas in explicitly judgment-free zones or as integrated as working in tandem to implement the fruits of adolescents’ political imagination.

Importantly, this responsibility to students does not simply lay at the teachers’ feet. School leadership can also implement policies that facilitate this process. For example, high schools often have extracurricular clubs housed within and approved by school leadership. Schools can provide needed funds and, if need be administrative support to around a school club that provides Black youth a safe space to process these ideas (this especially important in economically depressed areas in which Black youth have limited safe spaces). School administrators can also invest in training for teachers and school personnel around key concepts such as socio-political development theory and relevant programming like youthtopias. This professional development would ideally extend past core subject teachers (e.g., reading, math, science, and social studies) and engage all adults who work with youth in the school building. Ideally, by equipping *all* adults with the pertinent practical and theoretical knowledge to help foster Black youth’s political imagination, everyone has a stake in the process.

#### Youth-serving organizations

Youth-serving organizations are often seen as optimal spaces for sociopolitical development and by extension political imagination (Ginwright, 2007). From mentoring programs to community centers to after-school programs and high school-to-college pipeline programs, youth-serving organizations are diverse in the aim and scope of services provided (Kirshner, 2008; Ngo et al., 2017; Walker et al., 2021). At any given time, these spaces are simultaneously the source of mentoring, leadership development, academic support, counseling and socioemotional support, movement, general community, a meal, and other necessities to youth wellbeing (Kirshner and Ginwright, 2012; DeBower et al., 2021). Given their established range with youth and their families, youth-serving organizations are commonly mentioned sites of resistance (Ginwright, 2007; Nicholas et al., 2019). However, the work of nurturing and facilitating imagination in the context of resistance is a constant (and sometimes unrealized) challenge in the tradition of resistance in youth-serving organizations (Kirshner and Ginwright, 2012; DeBower et al., 2021). To that end, we offer three interconnected recommendations for organizations serving and guiding Black youth in their sociopolitical development.

First, youth-serving organizations should focus on developing authentic youth leadership and direction. Many of these organizations already include models of leadership development as part of their curricula. When we say authentic, we are calling for leadership that is grounded in the principles, concerns, and goals as formed by Black youth. One of the challenges youth-serving organizations face are their agendas and curricula that may be of minimal interest to the youth they serve. Moving forward, innovation in these organizations can look like listening to youth instead of programming them—this call for leadership development looks like working with youth to develop their own direction and vision.

To support this development of leadership, second, youth-serving organizations can develop more activities that facilitate a suspension of disbelief. These activities involve a guided move from CSA to imagining an ideal future absent from oppression and full of hope. This requires a facilitation that prioritizes limitless collective and individual creativity over established rules. Finally, to actualize the first two recommendations, we recommend that youth-serving organizations constantly (re)expose youth to narratives of previous success. Paired with creativity and imagination that reject perceived barriers to liberation, previous narratives of success serve as possibility models with useful methodologies and epistemologies for Black youth’s liberation. Taken together, these recommendations are designed to encourage the development of Black youth’s vision as well as their activism.

#### Faith communities

Religion often is central to the formation of political ideologies (Pearson-Merkowitz and Gimpel, 2009), and faith communities have long served as sites of political socialization and engagement for Black people specifically (Smith, 2003; McClerking and McDaniel, 2005; Harris-Lacewell, 2007; Todd, 2011). Black Christian churches, for example, have been central to the formation of some notable civil rights leaders, such as Martin Luther King Jr. and Fannie Lou Hamer, both of whom began their political engagement in their youth. The Black Muslim community laid the foundation for leaders such as Malcolm X, Betty Shabazz, and Muhammad Ali to fight for the global liberation of Black people (Auston, 2017). Then, a recent literature review by Pearce et al. (2019) also affirms that spiritual formation is strongly related to adolescent moral development and political ideologies. Pearce et al. (2019) noted, however, that there is limited research directly connecting adolescents’ political activism to religion or religiosity, calling for more work in this area. Still, the relationship between religion, moral values, and political ideology gives sufficient ground for suggesting that faith communities and Black adolescents’ political imagination be further explored (see Dean and Andrews, 2016).

Youth ministries in Black churches stand and a key site for potential political formation for Black youth. As youth’s faith communities teach concepts of justice, freedom, safety, and community through their particular religious lenses, one can reasonably infer that this has a consequence for how youth envision communities, enact advocacy and resistance, and what they conceptualize as the *end* of those efforts (Verma and Maria, 2006; Todd, 2011). Thus, faith leaders serving the youth in their faith communities ought to design their programming and faith education curricula in ways that leverage the strong connection between cultural values, social responsibility, political vision, and their faith tradition (Junkin, 2002; Verma and Maria, 2006; Pearce et al., 2019; King et al., 2020). It seems reasonable to infer that political imagination is an outgrowth of youth’s eschatological vision and the hope found therein.

King et al. (2020) studied the relationship between religiosity and hope among Salvadorian adolescents who participated in the faith-based youth development organization, Compassion International. King et al. (2020) used a structural education model to examine how the levels of religiosity both directly and indirectly related to the youths’ hope for the future. Results indicated that higher levels of religiosity are found among youth supported by Compassion International and there were significant direct and indirect relationships between youth’s level of religiosity and youth’s hopefulness. This study has implications for SPD and political imagination among Black youth because it highlights the role that active participation in faith-based service activism has on youths’ political outlooks and the moral convictions governing their engagement (also see Smith, 2003; Ginwright, 2011).

## Implications for future research

Watts et al. (2003) assert, “SPD is not limited to resisting oppression in the interest of justice, however; the capacity to envision and help create a just society is an essential part of the process as well” (p. 185). Studying Black adolescents’ political imagination is significant because their imaginations have served as the source of momentum for the revolutions that lead to social progress, the fuel of social justice (Turner, 2018; Hawlina et al., 2020). Based on our conceptualization of political imagination, we offer five considerations for scholars moving forward in this work: (1) prioritizing Black youths’ wellbeing; (2) honoring Black youths’ vulnerability in the imaginative process; (3) mindfully collaborating with Black youth in ways that advance their leadership and causes; (4) critically engaging the institutions and other stakeholders who impact this work; and (5) preserving opportunities for Black adolescents’ to experience the fullness of adolescence.

Though Black youth can (and often do) thrive, they do so in spite of endemic misrepresentation, marginalization, disenfranchisement, and dehumanization. When researchers request access to Black youths’ political imagination, the process or content therein, researchers are requesting access to what is often their only safe haven, their only fugitive space. Researchers also are asking Black youth to be emotionally vulnerable and engage in significant labor. As such, Black youths’ needs and wellbeing must be prioritized in this work. One of the reasons we struggle with the execution of collaborations or solidarity between youth and adult political actions is that we do not focus closely enough on the nature of alliance. Here collaboration, solidarity, and alliance should be understood as interrelated concepts. We do not take up the task of defining these concepts, but rather move to call for political engagement researchers to think more deeply about what collaboration looks like with Black adolescents when engaging in collective political action. To that end, a real-time understanding of what Black youth activists actually go through while doing and being so also is needed.

Citing YPAR as a common mode of studying Black adolescents’ sociopolitical development, researchers must guard against YPAR becoming another means of scientific voyeurism that fails to afford Black adolescents collaborative sociopolitical agency and dignity. This means the participatory research focusing on youth sociopolitical development must not only be youth-centered, but as much as possible, also youth-directed. Further, when we assert that Black youth should have sociopolitical agency, we also assert that their political interests are worthy of investment apart from the convergent interest of enfranchised adult co-conspirators (see Clay and Turner, 2021). Simply put: What are the youths’ concerns, goals, and desired approaches to resistance and reconstitution?

Finally, youth activism and political engagement researchers must (continue to) resist a grotesque proclivity to charge solely Black youth with redressing the immoral and inhumane systems and institutions spawned from the white political imagination, historically. This creates a quandary. Racism is the product of centuries of work interweaving white supremacist ideologies and reinforcing white dominance in every institution. Combating those systems and moving to dismantle those institutions is righteous and just. Yet, in efforts to upend racism and white supremacy, scholars may also conscript Black youth into another form of labor. This is a labor that potentially snuffs their childhood and adolescence. What does it mean for Black adolescence to be preoccupied with the atrocities inflicted by White adults? How can society better create space for Black youth to learn and practice resistance and envision a world free of these atrocities while also preserving/promoting/providing the *childness* of their childhood? Given the grave responsibility of this research, we also acknowledge the constraints of researchers who take up this work. As we push researchers to be just in their interactions with youth, to fully engage their stories and not exploit their labor, we also push the systems that regulate and support research activities. Simply put: We must hold funding agencies, promotion and tenure policies, advising and mentoring structures, academic journals and other power structures accountable for seeing, valuing, supporting, and protecting this research.

## Conclusion

This paper advances a developmental approach to understanding (a) political imagination, (b) its role in Black adolescents’ sociopolitical development, and (c) opportunities for adults, communities, and youth-serving organizations to effectively partner with Black youth. This research can support Black youth and their political mobilization by deepening our understanding of how one can cultivate and sustain Black adolescents as political visionaries and social justice leaders. Black youth have dreams of what their nation(s) ought to look like, and their dreams can inform how adults and organizations partner. The tasks of youth studies researchers, caregivers, educators, youthworkers, and even the youth themselves, is to push youth’s imaginations as far as it can see, and then create space for youth to apply the strategies that advance us toward that vision. Preparing Black youth to build a new world starts with childhood’s castle of Legos, Black history story time, and playing schoolhouse. As they mature—grow in their understanding, abilities, and desire to transform this complex world—Legos become letters of demand, story times become congressional hearings, and playing schoolhouse becomes organizing protest and teach-ins. The political imagination is used at each step.

## Author contributions

ES contributed to the conception and design of the manuscript as well as wrote the first draft of the manuscript. CS, JH, and LR wrote sections of the manuscript. All authors contributed to the article and approved the submitted version.

## Conflict of interest

The authors declare that the research was conducted in the absence of any commercial or financial relationships that could be construed as a potential conflict of interest.

The reviewer CM declared a shared affiliation to the CS to the handling editor at the time of the review.

## Publisher’s note

All claims expressed in this article are solely those of the authors and do not necessarily represent those of their affiliated organizations, or those of the publisher, the editors and the reviewers. Any product that may be evaluated in this article, or claim that may be made by its manufacturer, is not guaranteed or endorsed by the publisher.

## Footnotes

^1^Here, the term *politics* refers to the strategies and motives for organizing and influencing people, power, and resources for collective interests (Wiley, 2016).
