# Supplementary material for: Facing the rising sun: Political imagination in Black adolescents’ sociopolitical development
Source: Front Psychol. 2023 Feb 22;14:867749. doi: 10.3389/fpsyg.2023.867749 (PMC9994508; doi:10.3389/fpsyg.2023.867749)
Supplement: Supplementary file 1 [file Data_Sheet_1.docx]

**Appendix A**

**A Conceptual Model of the Paper’s Task**

| **Figure 1** |
| --- |
| *Paper Conceptual Map* |
| 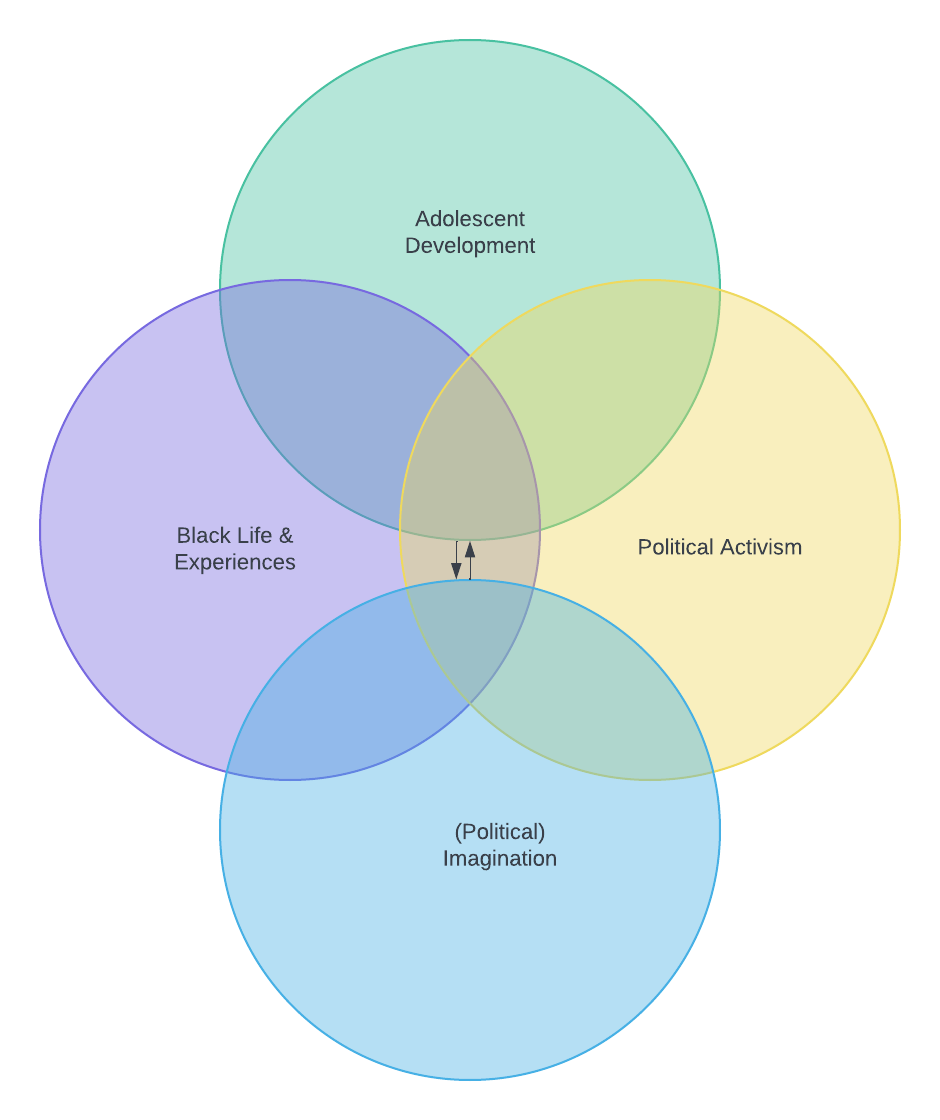 |
| *Note:* The image considers multiple disciplines to depict a conceptual understanding of this contribution and task, given the extant literature. Each of the circles represents a key area of the extant literature, many of which overlap, yet there remains a chasm in the literature regarding adolescence and imagination. The bidirectional arrows represent this papers section on imagination and adolescent development, which begins to address the chasm. Addressing the chasm is necessary in order to discuss Black adolescents’ political imagination within the context of SPD, which exists at the nexus all of circles overlapping. |

**Appendix B**

**Sociopolitical Development Model with Political Imagination Look Addition**

| **Figure 2** |
| --- |
| *Modified SPD Model* |
| 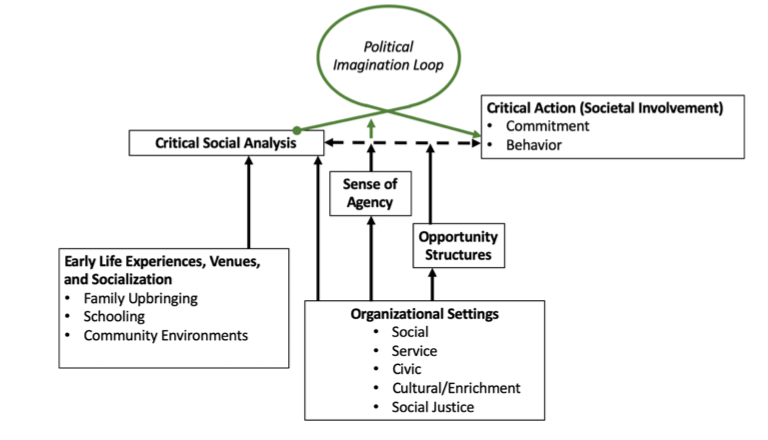*Note:* This image depicts the SPD model as presented by Watt and Guessous (2006), but with the addition of the political imagination loop – indicated in green. The circular point-end of the loop represents the rupture point where the person suspends presentism, determinism, and disbelief to begin the imaginative process. The triangular arrow-end is the moment of conscious return to the temporal present. The green arrow pointing from Sense of Agency to the loop indicates that agency is both exercised and developed in the imaginative process. |
|  |
